# Production of yeast cell wall polysaccharides‐β‐glucan and chitin by using food waste substrates: Biosynthesis, production, extraction, and purification methods

**DOI:** 10.1111/1541-4337.70161

**Published:** 2025-04-04

**Authors:** Deniz Günal‐Köroğlu, Gulsah Karabulut, Fariddudin Mohammadian, Aslı Can Karaca, Esra Capanoglu, Tuba Esatbeyoglu

**Affiliations:** ^1^ Department of Food Engineering, Faculty of Chemical and Metallurgical Engineering Istanbul Technical University Istanbul Türkiye; ^2^ Department of Food Engineering, Faculty of Food Engineering Sakarya University Sakarya Türkiye; ^3^ Department of Molecular Food Chemistry and Food Development, Institute of Food and One Health Gottfried Wilhelm Leibniz University Hannover Hannover Germany

**Keywords:** bioprocessing, dietary fiber, enzymatic extraction, prebiotics, *Saccharomyces cerevisiae*

## Abstract

Food waste causes significant environmental and economic challenges worldwide, prompting many nations to prioritize its reduction and recycling. As a nutrient‐rich material containing vitamins, proteins, and carbohydrates, it serves as a promising substrate for the cultivation of single‐cell microorganisms like yeast. Yeast cell wall polysaccharides (YCWPs), particularly chitin and β‐glucans, offer valuable applications in food, pharmaceuticals, and bioprocessing. This review highlights the biosynthesis, production, extraction, and purification of YCWP cultivated on food waste substrates. Key species including *Saccharomyces cerevisiae*, *Pichia pastoris*, and *Candida* spp. are discussed, with a focus on optimizing chitin and β‐glucan yield through mechanical, chemical, and enzymatic extraction methods. In addition, the structural and functional properties of β‐glucans and chitin in maintaining cell wall stability are explored, emphasizing their potential as prebiotics, dietary fibers, and biodegradable packaging materials. This review also examines the valorization of food waste in yeast cultivation, presenting a sustainable bioprocessing strategy for transforming waste into valuable bioproducts.

## 1 INTRODUCTION

The environmental and economic impact of food waste has led many countries to focus on its reduction and recycling (Salazar‐López et al., 2022). Understanding the production and extraction of yeast cell wall polysaccharides (YCWPs) from food waste is vital for biotechnology. The process involves yeast harvesting through filtration, centrifugation, or coagulation, followed by yeast cell wall disruption using physical, biological, or chemical methods to extract polysaccharides.

Various yeast species are utilized for this purpose, including *Kluyveromyces marxianus*, *Komagataella pastoris*, *Saccharomyces cerevisiae*, *Saccharomyces boulardii*, *Candida utilis*, *Pichia pastoris*, and *Wickerhamomyces anomalus* (Araújo, Ferreira, et al., 2020; Bzducha‐Wróbel et al., 2019; Divya et al., 2020; Farinha et al., 2019; M. Tang et al., 2020; Xing et al., 2018; Yuan et al., 2022).

β‐Glucans and chitin are essential components that contribute to the structural stability and function of cell walls in many organisms. β‐Glucans, particularly β‐1,3‐glucan with β‐1,6‐linked side chains, are known for their role in maintaining cell wall integrity, while chitin provides additional strength and protection. These polysaccharides help regulate the exchange of nutrients and waste within the cell. In the case of the YCWP, approximately 1200 genes are involved in the synthesis of these components, including *N*‐acetylglucosamine polymer, β‐1,3‐glucan, and β‐1,6‐glucan. The β‐1,3‐glucan molecules, forming a helical structure, provide both structural stability and permeability, which is crucial for nutrient uptake and waste removal (Chotigavin et al., 2021). Chitin, though less abundant, is crucial for structural integrity, concentrated in the inner cell wall and at bud scars, enhancing mechanical strength and protection, especially in pathogenic yeasts like *Candida albicans* (Garcia‐Rubio et al., 2020).

To access polysaccharides within the yeast cell wall, disruption methods must be chosen based on the composition, strength, and nature of the biomass. Among the widely used techniques, high‐pressure homogenization (HPH), ultrasonication, and the French press are particularly effective due to their ability to efficiently break down the cell wall while preserving polysaccharide integrity (Aazami et al., 2018; Bzducha‐Wróbel et al., 2019, 2020; Lu & Zhu, 2023). On the other hand, nonmechanical methods include high‐voltage electrical discharge, chemical methods, and enzymatic treatments (Gautério et al., 2023; Kot et al., 2020; N. Tang et al., 2022). Recent studies collectively draw attention to the impact of environmental and chemical factors on enhancing β‐glucan and chitin production and yeast cell wall properties, thereby emphasizing the need to optimize these conditions for improved yield and quality in industrial applications.

YCWPs, mainly β‐glucan and mannans, are widely used in the food industry for their ability to enhance texture and functionality (Caruso et al., 2022; Khan et al., 2022). In addition, YCWP provides significant health benefits, acting as anticancer and antidiabetic effects, while also offering immune modulation, cholesterol reduction, and improved gut health (Divya et al., 2020). Chitin and its derivative chitosan, known for its antimicrobial properties, are utilized in biodegradable food packaging materials, contributing to food preservation and sustainability (Priyadarshi & Rhim, 2020).

There has been growing interest in yeast cell wall compounds in recent years, with studies of structural features, biomedical applications, and bioactivities reviewed by Bastos et al. (2022), Liu, et al. (2021), and Jofre et al. (2024). However, gaps in the literature remain regarding the valorisation of food waste for yeast production and subsequent isolation of β‐glucan and chitin, particularly in developing of less aggressive extraction methods, optimizing environmental conditions to maximize yield, and addressing challenges associated with large‐scale industrial applications. Therefore, the β‐glucan and chitin referenced in this text include those sourced from the yeast cell wall. This review of YCWP provides significant insights into cellular biology and holds substantial industrial potential.

## 2 BIOSYNTHESIS OF YCWPs

Yeasts are unicellular organisms with a cell wall that plays a vital role in maintaining structural integrity and protecting against environmental stresses. Located outside the plasma membrane, the cell wall is primarily composed of proteins and polysaccharides, providing mechanical strength, regulating cell shape, and facilitating interactions with the environment. Its composition and structure can vary between yeast species, influenced by factors such as temperature, pH, and nutrient availability (Garcia‐Rubio et al., 2020). In general, yeast cell walls consist of 25%–30% polysaccharides by dry weight, with β‐glucans (29%–64%) as the dominant component, followed by mannans (31%), chitin (2%), and lipids (9%) (Liu et al., 2021).

The yeast cell wall contains both amorphous and polymeric polysaccharides, consisting of approximately 350 glucose monomers (Utama et al., 2023). The production and modification of yeast β‐(1,3)/(1,6)‐glucans are influenced by environmental conditions, nutrient availability, and species variations. These processes are regulated by stress‐related (Hog1) and cell integrity (Mkc1, Cek1) pathways (Bzducha‐Wróbel et al., 2024).

β‐1,3‐Glucan (55%) and β‐1,6‐glucan (12%) together account for a major portion (65%–90%) of the total β‐glucan content in the yeast cell wall (Chioru & Chirsanova, 2023). These glucans form a helical structure that is essential for maintaining cell wall integrity, strength, and permeability (Hamidi et al., 2022). Their structural and functional properties depend on the type of glycosidic bonds present (Y. Sun, Shi, et al., 2019). β‐1,3‐Glucans form the core framework of the inner cell wall layer, creating covalent linkages that connect other cell wall components (Pitarch et al., 2008).

The synthesis of these glucans is driven by specific enzymes, particularly those from the *FKS* gene family, which catalyze the polymerization of uridine diphosphate glucose (UDP‐glucose) into β‐1,3‐glucan chains. This process is regulated by the Rho1 GTPase signaling pathway (Hu et al., 2023; Klis et al., 2006). The *FKS* *gene* belongs to the GT48 glycosyltransferase family, though its precise function is still not fully understood. Ongoing research aims to further explore glucan biosynthesis and optimize yeast growth conditions (Bzducha‐Wróbel et al., 2024).

**FIGURE 1:**
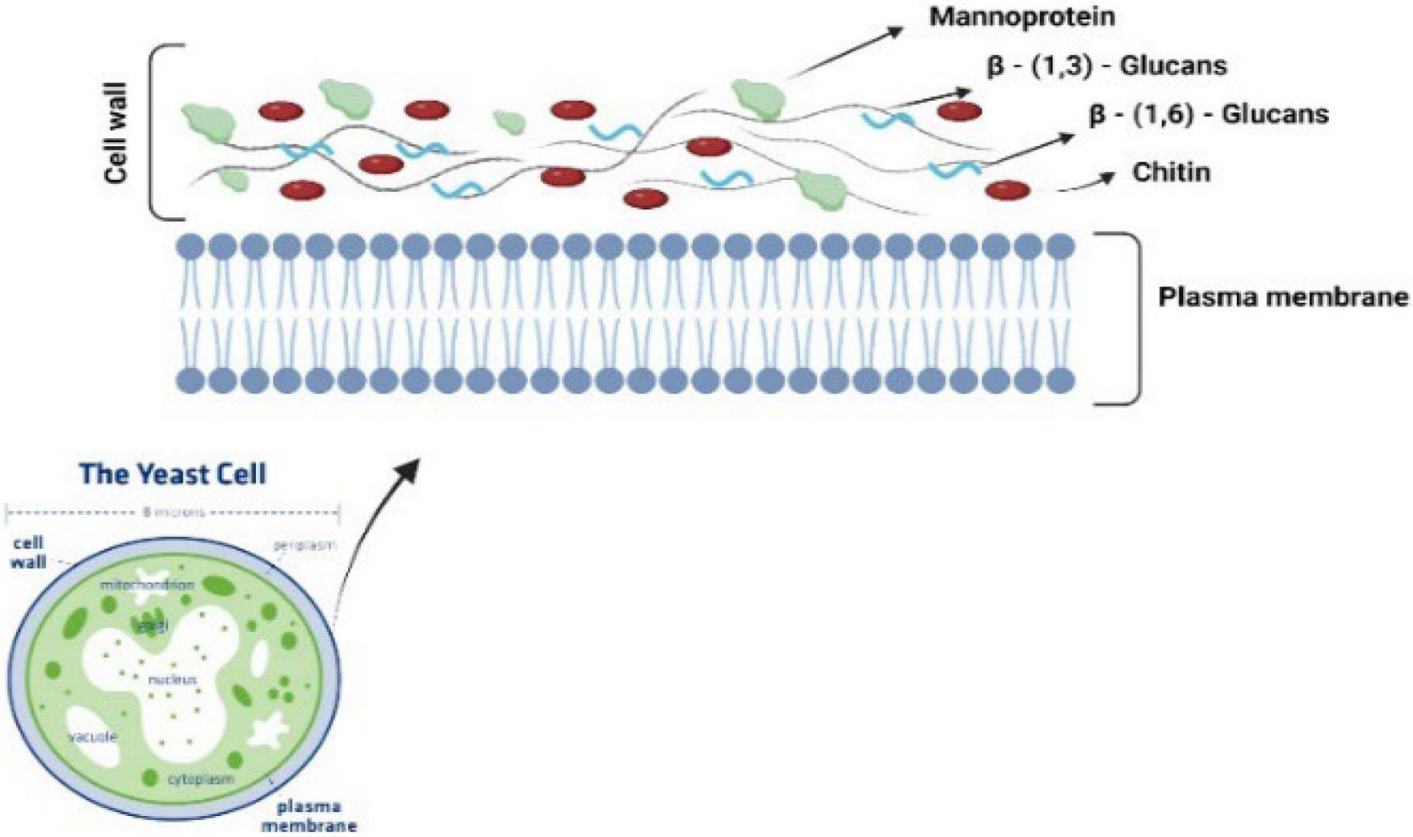
Yeast cell wall structure.

Although the composition of the yeast cell wall varies between species, *S. cerevisiae* has been extensively studied due to its widespread industrial and scientific applications. In *S. cerevisiae*, the cell wall consists of two main layers: an inner layer composed of chitin and glucans (primarily β‐1,3‐glucan and β‐1,6‐glucan) and an outer layer predominantly made up of mannoproteins (Figure 1). The glucans in the inner layer form a strong, dynamic network by covalently linking to other cell wall components, ensuring cell stability (Patel & Free, 2019).

While the biosynthesis of β‐glucans and chitin in *S. cerevisiae* is well documented, similar processes occur in other yeast species, with differences in the genes and enzymes involved. Yeast species such as *C. utilis* (Yuan et al., 2022), *K. marxianus* (Tang et al., 2022), and *P. pastoris* (Xing et al., 2018) also produce β‐glucans and chitin, but their cell wall composition and organization differ based on environmental adaptations. In fungi beyond *S. cerevisiae*, the genes responsible for β‐glucan production vary by species. For example, *Schizosaccharomyces* species regulate β‐glucan synthesis through alternative pathways, while *Candida* species possess additional enzymes that enhance cell wall flexibility and resistance to stress. Some yeasts also use distinct glycosylphosphatidylinositol‐anchored proteins for glucan crosslinking, allowing them to adapt their cell walls to different environmental conditions. These variations reflect evolutionary adaptations that influence fungal structure and survival (Lozančić et al., 2021).

β‐Glucan synthesis occurs in the plasma membrane with the involvement of proteins from the Golgi apparatus and endoplasmic reticulum; however, the precise mechanism of synthesis remains unclear. As shown in Table 1, several genes in *S. cerevisiae*, including Rot1, Skn1, Big1, Kre1, Knh1, Kre5, Kre6, and Kre9, are involved in β‐glucan biosynthesis (Byrne & Wolfe, 2005; Guillaume & Howard, 2006; Lozančić et al., 2021).

**TABLE 1:** Genes responsible for glucan and chitin synthesis in *S. cerevisiae*.

Gene	Polysaccharide synthesis	Activity	References
KRE1	β‐G	Acts as a glycoprotein in the assembly of β‐glucan in the cell wall	Boone et al. (1990)
KRE5	β‐G	A protein is involved in β‐1,6‐glucan synthesis	Meaden et al. (1990)
KRE6	β‐G	A glycosyltransferase plays a crucial role in the biosynthesis of β‐1,6‐glucan	Kurita et al. (2011)
KRE9	β‐G	Acts as a glycoprotein in the assembly of β‐glucan in the cell wall	Brown and Bussey (1993)
FKS1	β‐G	The addition of glucose units from UDP‐glucose to a growing glucan chain via the formation of β‐1,3 glycosidic linkages	Hu et al. (2023), Zhao et al. (2023)
FKS2	β‐G	Serves as the catalytic domain of the β‐1,3‐glucan synthase enzyme	Byrne and Wolfe (2005) Huang et al. 2005)
FKS3	β‐G	This protein is involved in the construction of the spore wall and shares structural and functional similarities with the catalytic subunits Gsc2p and Fks1p	Ishihara et al. (2007), Reinders et al. (2006)
RHO1	β‐G	Activates β‐1,3‐glucan synthase (Gsc2p and Fks1p) and is involved in cell wall biogenesis	Hu et al. (2023), Zhao et al. (2023)
BIG1	β‐G	The endoplasmic reticulum integral membrane protein is essential for maintaining normal levels of cell wall β‐1,6‐glucan	Azuma et al. (2002)
KNH1	β‐G	A Kre9p‐like protein; Kre9p is involved in cell wall β‐1,6‐glucan biosynthesis; increased production of this protein suppresses the growth defect of a Kre9‐deficient mutant; essential for propionic acid resistance	Mira et al. (2009)
SKN1	β‐G	A Type II transmembrane protein involved in sphingolipid biosynthesis; SKN1 is a paralog of KRE6, arising from a whole‐genome duplication event	Byrne and Wolfe (2005), Thevissen et al. (2005)
CHS1	Chitin	Chitin synthase I; the zymogenic form requires activation to catalyze the transfer of *N*‐acetylglucosamine (GlcNAc) to chitin	Ziman et al. (1996)
CHS2	Chitin	Chitin synthase II is an enzyme that, upon activation from its inactive precursor, catalyzes the polymerization of *N*‐acetylglucosamine (GlcNAc) to form chitin. This enzyme is indispensable for the synthesis of chitin in the primary septum during cell division	Oh et al. (2012), Teh et al. (2009)
CHS3	Chitin	Chitin synthase III is an enzyme that catalyzes the polymerization of *N*‐acetylglucosamine (GlcNAc) to form chitin. This enzyme is indispensable for synthesizing the bulk of the cell wall's chitinous component, the chitin ring that forms during budding, and the chitosan of the spore wall	Starr et al. (2012)
CHS5	Chitin	A component of the exomer complex, which includes Bud7, Bch2p, and Csh6p, is involved in the export of Chs3p from the Golgi apparatus to the plasma membrane	Paczkowski et al. (2012), Trautwein et al. (2006)

β‐1,3‐Glucan plays a crucial role in maintaining the stability and strength of the yeast cell wall while also supporting cellular integrity (Guillaume & Howard, 2006). These glucans are synthesized in the plasma membrane by β‐1,3‐glucan synthases and are subsequently integrated into the cell wall structure (Klis et al., 2006). The *FKS* gene family encodes the catalytic subunits of β‐1,3‐glucan synthases, including Fks1p, Gsc2p/Fks2p, and Fks3p, which use UDP‐glucose as a substrate. During synthesis, glucose units are sequentially added to the growing glucan polymer, which is then extruded through the enzyme's channel and incorporated into the cell wall (Klis et al., 2006). Studies in *Neurospora crassa* have demonstrated that FKS proteins function as catalytic units of β‐1,3‐glucan synthases by directly binding to UDP‐glucose. Another important protein family involved in β‐1,3‐glucan remodeling is the Gas family, which consists of five members (Gas1p–Gas5p). These proteins contribute to glucan modification and restructuring, playing a key role in maintaining the dynamic properties of the cell wall (Mouyna et al., 2000; Popolo & Vai, 1999).

Chitin is another essential component of the yeast cell wall, contributing to its structural integrity. Disruptions in chitin synthesis can lead to alterations in cell membrane shape and compromise cell wall stability (Brauer et al., 2023). Chitin chains are linked by hydrogen bonds in an antiparallel arrangement, forming a rigid structure. These linkages occur through the connection of the reducing end of chitin to the nonreducing end of glucose within the β‐1,3‐glucan polymer, further reinforcing the strength and resilience of the yeast cell wall (Patel & Free, 2019).

Although chitin is less abundant than other cell wall components, it plays a crucial role in maintaining the structural integrity and rigidity of the yeast cell wall. It forms a network of fibers linked by hydrogen bonds in an antiparallel arrangement, strengthening the wall and preserving its shape. The synthesis and regulation of chitin involve several enzymes, particularly chitinases such as CS1, CS2, and CS3, which participate in assembling and repairing the cell wall during the yeast cell cycle (Lenardon et al., 2010; Orlean, 2012).

In *S. cerevisiae*, chitin production is controlled by the cell wall integrity pathway, which responds to environmental stress and cell wall damage. The key regulator, Rho1, activates protein kinase C (Pkc1) and a MAPK cascade, leading to the activation of transcription factors Rlm1 and SBF. These factors regulate genes involved in cell wall synthesis, including GFA1, which increases the production of the chitin precursor GlcNAc (Nguyen et al., 2025). In addition, CHS3, the primary chitin synthase enzyme, is mobilized to the plasma membrane for chitin synthesis, while CHS7 aids in transporting Chs3, especially when β‐glucan synthesis is compromised. Although some studies suggest that Rlm1 may directly regulate CHS3, the evidence remains inconsistent (Nguyen et al., 2025).

Chitin synthesis must be carefully regulated in both time and space during the cell cycle. Class II enzymes like ScChs2 produce the primary septum, whereas Class I enzymes like ScChs1 function as repair enzymes, filling chitin into the birth scar or bud site after cytokinesis (Lenardon et al., 2010). Research by Nguyen et al. (2025) suggests that mutations in key cell wall integrity pathway genes (RHO1Q68H and PKC1R398A) can serve as genetic switches to enhance chitin production under stress conditions. Notably, 80%–90% of chitin synthesis is attributed to Chs3, which originates in the endoplasmic reticulum but becomes active in the plasma membrane (Orlean, 2012; Valdivieso et al., 1991).

The biosynthesis of β‐glucan and chitin in *S. cerevisiae* involves several specialized enzymes. EXG1 encodes exo‐1,3‐β‐glucanase, a crucial enzyme for cell wall integrity and β‐glucan synthesis (Cappellaro et al., 1998). Similarly, SPR1 encodes another exo‐1,3‐β‐glucanase, which is essential for heat resistance in ascospores and plays a role in sporulation (Muthukumar et al., 1993). BGL2 produces endo‐β‐1,3‐glucanase, which maintains cell wall structure and facilitates the attachment of mannoproteins (Plotnikova et al., 2006).

For chitin processing, CTS1 encodes endochitinase, which is necessary for cytokinesis and cell separation (O'Conallain et al., 1999), while CTS2 produces both exochitinase and endochitinase, playing a compensatory role in sporulation—especially in *Aspergillus gossypii*—as part of the GH18 glycoside hydrolase family (Jiang & Yan, 2023). In addition, CRH1 and UTR2 encode chitin transglycosylases, which link chitin to β‐1,6‐glucan and β‐1,3‐glucan, further strengthening the yeast cell wall (Cabib, 2009; Cabib et al., 2008). These enzymes work in coordination to ensure the structural integrity and functionality of the yeast cell wall.

## 3 PRODUCTION OF YCWPs

With the growing global population, food waste has significantly increased, posing challenges to food security, environmental sustainability, and waste management (Sagar et al., 2024). Annually, 1.3 billion tonnes of food are wasted, including dairy products, cereals, seafood, meat, beverages, vegetable and animal oils, and other categories (Ansari et al., 2024; Van et al., 2022). Utilizing food waste as a substrate for microbial fermentation offers a sustainable solution by converting these residues into valuable products, such as YCWPs, which have applications in food, pharmaceuticals, and biotechnology.

Food waste‐derived lignocellulosic biomass, including agricultural residues and processing byproducts, serves as a rich source of carbohydrates for yeast cultivation (Piercy et al., 2024). However, the high lignin content and recalcitrance in some food wastes require pretreatment strategies such as enzymatic hydrolysis, acid hydrolysis, or alkaline treatment to improve digestibility and enhance sugar availability for yeast fermentation (Wan et al., 2023). Sugarcane bagasse, a major agro‐industrial byproduct, has been successfully utilized for yeast cultivation. Dos Santos Nascimento et al. (2022) demonstrated that *Candida robusta* URM5293 produced 141 g/L of biomass using alkaline‐hydrolyzed sugarcane bagasse under optimized conditions, highlighting its potential for yeast cell wall production.

Molasses, a byproduct of sugar production, contains high levels of fermentable sugars such as glucose, fructose, and sucrose, making it an excellent substrate for yeast fermentation. Hashem et al. (2022) found that *Hanseniaspora guilliermondii* and *Issatchenkia orientalis* efficiently utilized date molasses, achieving high biomass yields under optimal conditions (30°C, pH 4) with peptone supplementation enhancing protein and YCWP production.

The efficiency of different food waste sources in YCWP production depends on substrate composition, microbial strain selection, and fermentation conditions. Gozan et al. (2023) reported that enzymatic hydrolysis of arrowroot and cassava waste yielded high glucose conversion rates (95.93% and 64.70%, respectively), with nitrogen supplementation, like peptone, further enhancing β‐glucan production. Dewi et al. (2021) determined that optimizing substrate composition using tofu waste, bran, fish meal, and molasses resulted in superior β‐glucan yields (25.9 g/kg waste), emphasizing the importance of nutrient balance in fermentation media.

Dairy waste, particularly whey, has been explored as a substrate due to its high protein and lactose content, making it a valuable substrate for microbial fermentation. Vasilakis et al. (2022) reported that *Papiliotrema laurentii* NRRL Y‐2536 produced a “sticky” and “gummy” aggregate composed of yeast biomass and extracellular polysaccharides during fermentation via mizithra second cheese whey. Similarly, Koukoumaki et al. (2024) demonstrated that *K. marxianus* strain EXF‐5288 achieved optimal growth using deproteinized cheese whey, leading to high biomass yields (9.40 g/L). These findings highlight the potential of dairy waste for YCWP production.

Yeast cell wall components, primarily β‐glucans and mannans, are valuable bioactive compounds with applications in food, pharmaceuticals, and animal feed (Baek et al., 2024). The production process involves several key stages: substrate selection, yeast cultivation, fermentation optimization, biomass harvesting, and polysaccharide extraction (Figure 1). Utilizing food waste as a substrate offers a sustainable and cost‐effective alternative to conventional carbon sources, reducing environmental impact while enhancing economic feasibility (Abdullahi et al., 2021).

Different food waste sources vary in their efficiency for YCWP production. For example, potato peel has been shown to enhance yeast growth due to its high starch and carbohydrate content, resulting in increased polysaccharide yields (Khan et al., 2022). In contrast, citrus waste contains antimicrobial compounds such as limonene, which can inhibit yeast growth unless pre‐treatment methods such as autoclaving or enzymatic hydrolysis are applied (Gervasi et al., 2018). The composition of the substrate significantly influences the final yield and structure of the extracted polysaccharides.

**FIGURE 2:**
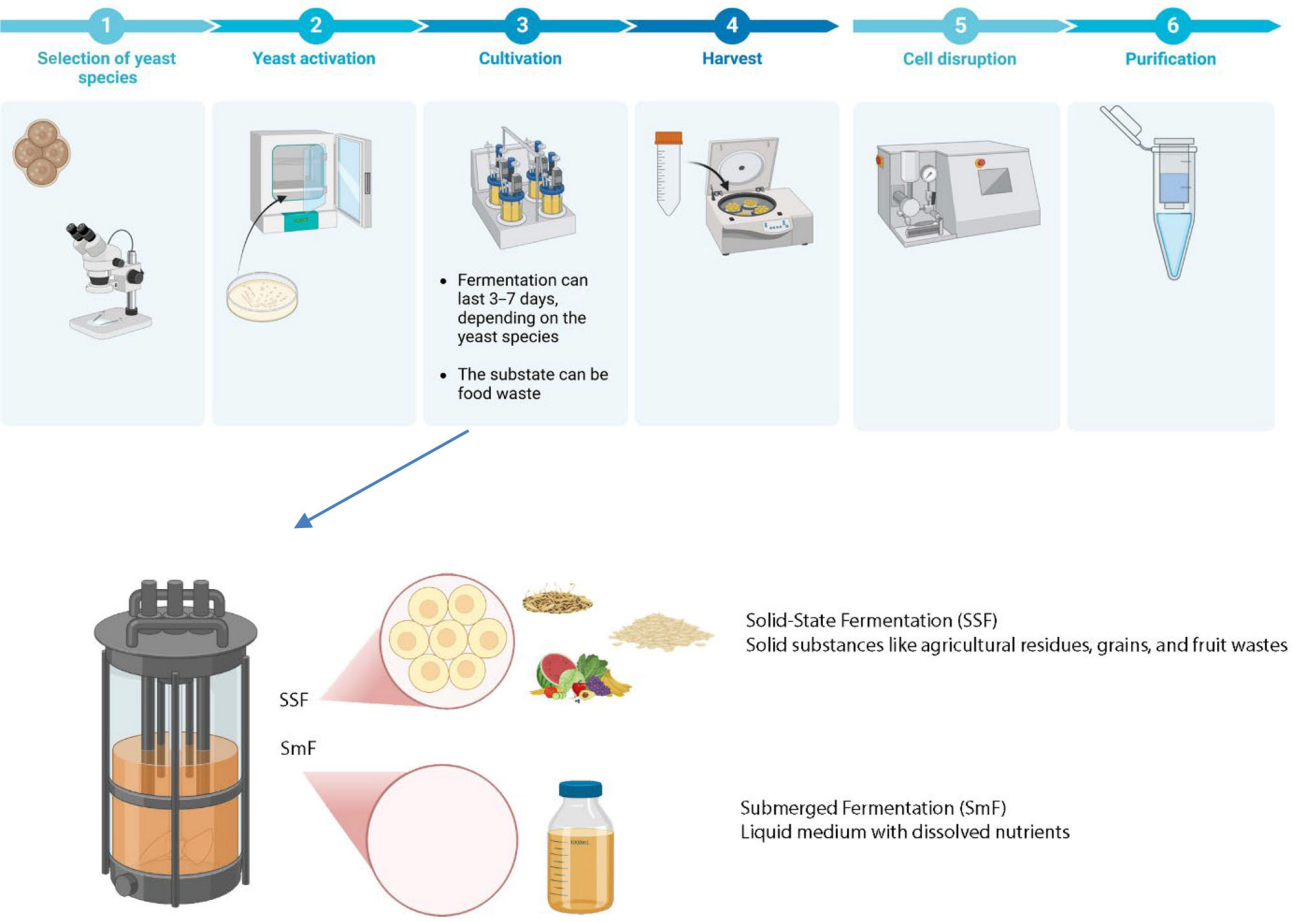
Yeast cell production from food waste.

Solid‐state fermentation (SSF) and submerged fermentation (SmF) are the two primary methods employed in yeast cultivation (Figure 2, Step 3). SSF, often used with dry agricultural residues, supports higher polysaccharide production due to enhanced cell wall synthesis under stress conditions (Chilakamarry et al., 2022). On the other hand, SmF is more suitable for moisture‐rich substrates like fruit and vegetable waste, offering higher biomass yields but requiring more intensive downstream processing (Carranza‐Méndez et al., 2022). Comparing these methods, SSF often leads to more structurally complex polysaccharides with enhanced functional properties, while SmF provides higher overall productivity (Shakouri et al., 2023).

Once fermentation is complete, the microbial biomass is harvested through centrifugation or filtration, followed by drying methods such as freeze‐drying or spray‐drying to preserve polysaccharide integrity (Aidoo et al., 2023). Extraction processes, including alkali or enzymatic treatments, are then applied to isolate β‐glucans and mannans, ensuring maximum yield and bioactivity (Thiviya et al., 2022).

Optimizing food waste utilization and fermentation conditions can boost both the yield and functionality of YCWPs. Future research should refine substrate formulations and develop affordable pretreatment methods to improve industrial efficiency. Using food waste as a sustainable resource reduces environmental impact and supports circular economy efforts. To maximize YCWP production, it is crucial to tailor fermentation strategies based on waste substrates and optimize bioprocess parameters. Exploring new microbial strains may further enhance YCWP production from diverse food waste sources.

## 4 EXTRACTION METHODS

**FIGURE 3:**
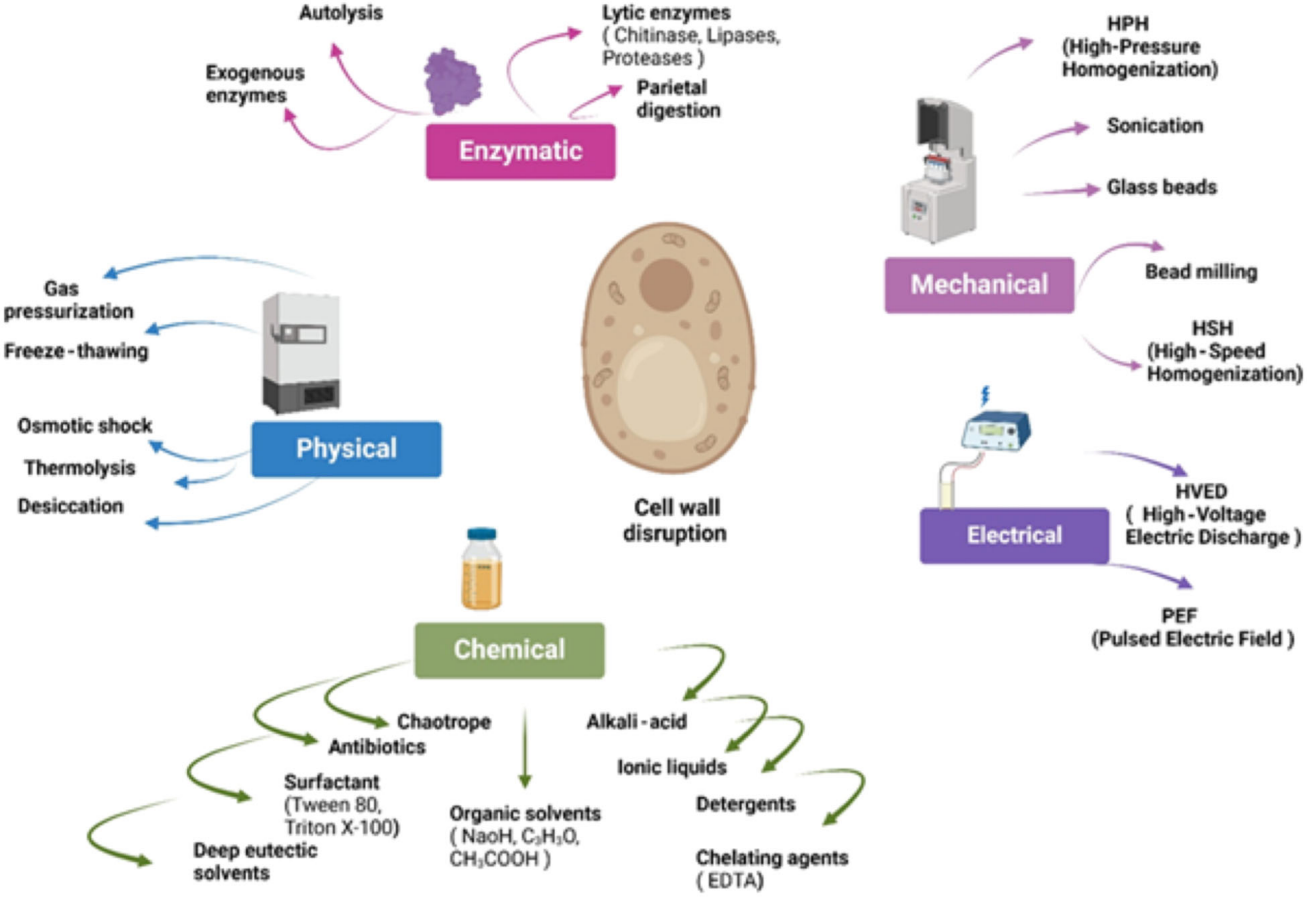
Different methods of yeast cell wall disruption.

In order to access the polysaccharides within the yeast cell wall, appropriate degradation and extraction methods must be selected based on the composition, strength, and nature of the biomass, necessitating the breaking of the cell wall (Amer et al., 2021; Kot et al., 2020). Also, the selected method should be a high‐productivity method that not only maintains the biological activity of the yeast but also should not produce cracked cells during the disruption process (Amer et al., 2021). As shown in Figure 3, YCWP extraction methods are divided into two main groups: i) mechanical and ii) nonmechanical methods. Table 2 shows the recent extraction methods of varying YCWPs and the main findings.

**TABLE 2:** The recent extraction methods of varying yeast cell wall polysaccharides and the main findings.

YCWPs	Yeast species	Growth media	Extraction methods	Main findings	References
Chitin/chitosan	*S. cerevisiae*	Nutrient yeast dextrose broth	Alkaline extraction, followed by acid extraction	The chitin content of the sample: 92.80%	C. Sun et al. (2018)
Chitin–glucan complex	*Komagataella pastoris (Pichia pastoris)*	Medium K	Alkaline extraction (NaOH 5 M, 65°C, 2 h) Neutralized Dialysed 12,000 kDa	Chitin–glucan complex: 19% of the biomass	Farinha et al. (2019)
Chitin–glucan complex	*Komagataella pastoris*	Glycerol	Hot alkaline extraction (NaOH 5 mol/L, 65°C, 2 h) Neutralized	Chitin–glucan complex: 20% of the cell dry mass	Araújo, Alves, Lima, et al. (2020)
Chitin	12 yeast species (*Candida* sp., *Clavispora* sp., *Nakaseomyces* sp., *Pichia* sp., *Saccharomyces* sp.)	Synthetic complete medium	Lysis buffer (50 mM Tris, 50 mM EDTA, 2% SDS, and 40 mM β‐mercapto‐ethanol) for 1 h at 100°C Sterile saline solution and ultrasonication 4×, 45 s SDS buffer (50 mM Tris, pH 8; 100 mM EDTA, pH 8; 2% SDS) at 100°C	Chitin patterns and morphology were observed	Aldebert et al. (2024)
β‐Glucan	*S. cerevisiae*	Sterilized, deionized water	High‐pressure homogenization (HPH) (200–600 bar, 1–3 passes) Autolysis (cylindrical glass containers (6.5 cm × 12 cm, 48 h)	↑Yeast autolysis and total solids yield with HPH treatment ↑The β‐glucan content of the solid autolysis residue with increasing treatment intensity and autolysis time by up to 100%	Dimopoulos et al. (2020)
β‐Glucan	*Candida guilliermondii*, *Candida lusitaniae*, *Candida parapsilosis*, *Pichia pastoris*, *Saccharomyces pastorianus*, and *S. cerevisiae*	YPG broth	Hot water extraction method Autoclaved Ultrasound (20 kHz, 240 W, 30: 10 s on: off, 15 min) Lipid removal with isopropyl alcohol	↑Protein content of β‐glucan obtained from *C. guilliermondii*, *P. pastoris*, and *S. pastorianus* β‐G/cell wall mass: 37.26 (*Candida lusitaniae*)‐57.78% (*Saccharomyces pastorianus*)	Bikmurzin et al. (2022)
β‐Glucan	*S. cerevisiae*	Banana waste, papaya waste, and napa cabbage waste	Cell autolysis (15 min, 80°C) Alkaline extraction Alkaline extraction (1.0 M NaOH, 2 h, 80°C) Washing (1.0 M CH_3_ COOH, 2 h, 80°C)	The highest β‐G: 9.094 g in papaya waste	Utama et al. (2020)
β‐Glucan	*Candida albicans SC5314*, *C. dubliniensis*, *C. glabrata*, and *C. haemulonii*	YPD broth (1% yeast extract, 2% peptone, 2% dextrose)	Alkaline extraction 0.1 N NaOH (15 min at 100°C) Neutralization to pH 7.0 Extracted with 1.0 N H_3_PO_4_ (15 min at 100°C) Neutralized to pH 7.0, Lipid removal (boiling absolute ethanol, 15 min)	β‐Glucan and glycogen form a novel macromolecular complex in the cell wall	Lowman et al. (2021)
β‐Glucan	*S. cerevisiae*	YPD broth (1% yeast extract, 2% peptone, 2% dextrose)	Alkaline extraction 2% sodium hydroxide for 5 h at 90°C Neutralization Ethanol precipitation	XRD‐the presence of β‐glucan molecules at a peak at 29.481°	Divya et al. (2020)
YCWPs	*Saccharomyces boulardii*		Ultrasound‐assisted extraction 52.63% NaOH addition, 143.15 W ultrasonic power, and 86.20 min Purification DEAE‐52 Sepharose Fast Flow exchange column and Sephadex G‐100 column	↑Extraction yield with ultrasound treatment time from 30 to 80 min ↑Extraction yield with NaOH addition up to 60 g/L ↑Extraction yield with ultrasound power up to 150 W Max yield of YCWP: 37.54%	M. Liu, Liu, et al. (2021)
β‐(1,3)/(1,6)‐Glucan	*Candida utilis*	Deproteinated potato juice water with 10% (w/v) of pure glycerol	Bead milling (Zirconium‐glass beads of 1 mm in diameter) in 0.02 M sodium potassium buffer, pH 7.5 Autoclaved (121°C/0.1 MPa/30 min) Isopropyl alcohol 60°C, 2 h Protein removal Pronase E enzyme 37°C for 24 h	Purification of β‐(1,3)/(1,6)‐glucan: 85%	Bzducha‐Wróbel et al. (2019)
β‐(1,3)/(1,6)‐Glucan	*Candida utilis*	Deproteinated potato juice water with 10% (w/v) of pure glycerol	Treatment 1 Alkaline‐acid treatment with hot‐water extraction (1 M NaOH, 90°C/1 h/ autoclaving 121°C/0.1 MPa/1 h) Treatment 2 Isolation based on cell wall autoclaving, lipids extraction, and proteolysis (Zirconium‐glass beads of 1 mm/ autoclaved 121°C, 0.1 Mpa, 1 h/ Isopropyl alcohol 60°C, 2 h/ Pronase E enzyme 37°C for 24 h)	β‐(1,3)/(1,6)‐glucan extraction yield for both methods: 82% ↑β‐(1,3)/(1,6)‐glucan ratio for Treatment 2 ↑Production efficiency of 70%, compared to 49% for Treatment 1 The ratio of alkali‐insoluble β–(1,3)/(1,6)‐glucan: Treatment 2 (1.23) > Treatment (1.04)	Bzducha‐Wróbel et al. (2020)
β‐Glucan	*Pichia pastoris*		Autolysis (w: v, 1: 15, pH6, 50°C, 30 h, NaCl) High‐pressure hot‐water treatment (w: v, 1:30, pH7.5, 4 h) Ultrasound (w: v, 1: 40, 400 W, 20 kHz, 10s, 60 cycle), Isopropanol extraction (5 x, v:v, 1:4, 85°C, 2 h) Protease treatment (pepsin, papain, flavoenzyme, trypsin, bromelain, and thermoysin, 4 h) Purification Dialysis 13,000 kDa	β‐glucan purity: 85.3% β‐glucan yield: 11.7% ↑Autolysis with NaCl ↓Ash, genetic material, water‐soluble mannan, and mannoproteins with Hot water treatment ↑Release of β‐1,6‐Glucan with ultrasound treatmen	Xing et al. (2018)
β‐Glucan	*S. cerevisiae*	Commercial crude β‑G	Ionic liquid extraction 1‐butyl‐3‐methyl‐imidazolium chloride (80°C, 30 min) Precipitated with adding water Purification Filtration and washing with hot water (3x)	β‐Glucan purity: 95.2% β‐Glucan yield: 83.4% β‐1,3‐Glucan content: 78.2% with 98.4% purity.	Pham et al. (2020)
β‐Glucan	*Saccharomyces carlsbergensis*	Beer beverage waste	Alkaline extraction (6% w/v NaOH at 90°C for 2 h) Acid precipitation pH 7	↑ β‐Glucan content up to 42.23% with tannic acid addition to the media	Chotigavin et al. (2021)
β‐Glucan	*S. cerevisiae*	Commercial crude β‑G	Alkali extraction and acidolysis 2.0 M NaOH, 4°C for 12 h Degradation of the insoluble residue with 1.0 M HCl/ H_2_SO_4_ Purification Dialysis 8000–14000 Da Size exclusion chromatography Sephacryl S‐400 column	β‐Glucan yields by alkali extraction and acidolysis were 12.41% and 42.85%	Zheng, Huang, and Ling (2019)
β‐Glucan	*S. cerevisiae*	Ultrapure water	Acid‐base extraction Water extraction	↑Biological efficacy of β‐Glucan of acid‐base extracted than in the water extraction method	(Amer et al., 2021)
β‐1,3‐Glucan	*S. cerevisiae*	NYDA plate	Mechanical vibration in a tissue grinder Alkaline extraction 6 h 75°C with 3% NaOH Acidic extractions 0.5 M acetic acid for 3 h at 90°C	↑95.65% β‐(1,3)‐Glucan/cell wall mass The molecular weight of β‐(1,3)‐ Glucan: 165 kDa	(C. Sun et al., 2019)
β‐Glucan	*S. cerevisiae*	Spent brewer's yeast slurry	Alkali Treatment combined with high‐Pressure 0.85% alkali concentration, 6.5:1 liquid‐solid ratio, 108°C temperature (pressure, 0.039 MPa), and 5 min	β‐Glucan content and extraction rate: 78.11 and 78.38% ↓Alkali dosage and processing time with High‐pressure treatment and alkali treatment	(Tian et al., 2019)
β‐Glucan	*S. boulardii RC009*, *S. cerevisiae RC012* and *S. cerevisiae VM014*	Dry distillery grain soluble extract or yeast extract‐peptone‐dextrose broth	Glass bead milling (0.45 mm) Mechanical shaking 4°C, 30 s, 5x Centrifugation	↑Thickness of the cell wall using dry distillery grain soluble extract	(Pereyra et al., 2018)
β‐Glucan	*Saccharomyces pastorianus*	The spent yeast biomass	Autolysis (50°C for 24 h) Alkaline extraction at 80 °C for 2 h Acid extraction 75 °C for 1 h	β‐Glucan content: 19.79 mg/100 g dw	(Martins et al., 2018)
β‐Glucan	*Candida utilis*		High‐pressure steam synergistic enzymatic extraction Glass bead milling (0.3 and 0.4 cm of diameter, solid‐liquid ratio of 10 %) High‐pressure steam treatment at 121°C for 2 h 2% neutral protease and papain Ultrasound‐assisted cell crash Dialyzed for 48 h	82.65 % β‐Glucan content ↑The solubility of yeast β‐Glucan with Ultrasound‐assisted H_2_O_2_ from 13.60 to 70.00 ↓Molecular weight from 6.73 × 106 Da to 1.22 × 106 Da ↑Free radical scavenging activity	(Ma et al., 2022)
Modification					
β‐Glucan	*S. cerevisiae*		Acid degradation treatment on crude β‐G 45% sulfuric acid treatment at 20°C incubated at 10°C, 20°C, or 30°C for 1 h, 3 h, 6 h, or 12 h neutralized with 13 N NaOH Dialyzed	↑Solubility ↑Low‐molecular‐weight soluble β‐Glucan with higher purity.	(Ishimoto et al., 2018)
β‐Glucan	*S. cerevisiae*		Ultrasound‐ and alkali‐assisted enzymolysis Crude β‐G Ultrasound pre‐treatment 20 kHz and 420 W The solid‐liquid ratio of 1:1000 g/mL the treatment time of 1 h, and the volume of 300 mL Alkali pre‐treatment 2 M NaOH at 4°C for 12 h Enzyme treatment Viscozyme L, 50°C, pH 5.0, 60.5 FBG/g, 1.5 h Purification Anion exchange chromatography resin column	↓The particle size of yeast β‐Glucan with ultrasound pre‐treatment from 8.80 µm to 1.77 µm; with alkali pretreatment to 7.19 µm ↑Water soluble β‐Glucan yield to 32.3% and 36.2% for ultrasound and alkali treatments ↑The purity of β‐Glucan reached 98.8% after zymoprotein removal	Zheng, Huang, Luo, et al. (2019)
β‐1,3‐Glucan	*S. cerevisiae*		Phosphorylation Purification Ion‐exchange chromatography column	↑Antioxidant activities in vivo	Mei et al. (2020)
β‐Glucan	*Candida utilis*		β‐Glucanase enzyme treatment Ultrasound intensity (10%–80%)	↑The solubility of yeast β‐glucan: 75.35% ↑Antioxidant activity	Yuan et al. (2022)

Abbreviations: dw: dry weight; XRD: X‐ray powder diffraction; YCWPs: yeast cell wall polysaccharides.

### 4.1 Mechanical Extraction

Mechanical extraction techniques such as bead milling, homogenization, and ultrasonication for isolating polysaccharides from yeast cell wall primarily involve mechanically disrupting the yeast cells. This disruption releases the intracellular polysaccharides, allowing them to be further processed and purified (M. Liu, Liu, et al., 2021).

Milling techniques such as ball milling with small beads (e.g., glass or zirconium), involve placing yeast cells in a rotating cylinder with hard material balls, which collide with the cells, leading to their disruption (Aazami et al., 2018; Bzducha‐Wróbel et al., 2019, 2020). In the zirconium‐glass beads sizes of 0.45–4 mm mechanical method, the suspension is mixed with glass beads, then the resulting suspension is centrifuged for separation (Ma et al., 2022; Pereyra et al., 2018). Bzducha‐Wróbel et al. (2019) extracted water‐insoluble yeast cell wall and purified yeast β‐glucan from *C. utilis* ATCC 9950 following biomass cultivation using waste potato juice water and glycerol. The yeast cell wall was separated from the biomass through bead milling (zirconium‐glass beads of 1 mm in diameter), followed by isopropyl alcohol treatment, enzymatic protein hydrolysis, and autoclaving, resulting in 85% β‐(1,3)/(1,6)‐glucan.

Cryogrinding involves freezing the yeast cells with liquid nitrogen before grinding them into a fine powder. The extreme cold makes the cell walls brittle, facilitating their breakage during grinding. Freeze‐thaw cycling is a gentler method, which repeatedly freezes and thaws the yeast cells (Lu & Zhu, 2023). The formation and melting of ice crystals within the cells cause mechanical damage to the cell walls, aiding in the release of polysaccharides (C. Sun, Jin, et al., 2019).

HPH forces cells through a narrow valve at high pressure, generating shear stress and cavitation that disrupts the cell walls. Dimopoulos et al. (2020) found that HPH (200–600 bar with 1–3 passes) significantly increased the β‐glucan content of *S. cerevisiae* autolysis residue by up to 100%, enhancing yields of protein, amino acids, and total solids. Higher treatment intensity and longer autolysis times further increased β‐glucan content while decreasing protein content. Tian et al. (2019) showed that combining alkali treatment with high pressure (0.039 MPa) substantially reduces alkali dosage and extraction time, achieving 78.11% β‐glucan content and 78.38% extraction yield. High‐pressure hot‐water treatment serves to eliminate ash, genetic material, and water‐soluble polysaccharides, ensuring a cleaner extract (Xing et al., 2018). Subsequent steps include isopropanol extraction and protease treatment to further purify the extract. The extraction and purification of β‐glucan from *P. pastoris* results in an 85.3% purity and an 11.7% yield (Xing et al., 2018).

Ultrasonication is another widely used technique, utilizing ultrasonic waves to create rapid pressure changes in the medium (Aazami et al., 2018). Cavitation involves the rapid nucleation, growth, and collapse of micrometer‐scale bubbles (Ma et al., 2022). The collapse of these bubbles generates shear forces, accompanied by instantaneously high temperatures and pressures, and forms shockwaves that intensify interparticle collisions (Karabulut & Feng, 2024). Ultrasonication and alkali‐assisted enzymolysis significantly reduced the particle size of yeast β‐glucan, with ultrasound‐induced cavitation disrupting aggregates and exposing internal structures, enhancing enzymolysis efficiency and water‐soluble yeast β‐glucan yield (Zheng et al., 2019). Liu et al. (2021) reported that alkali‐assisted ultrasonic treatment increased *S. boulardii* YCWP extraction yield rapidly from 30 to 80 min and power up to 150 W but stabilized with longer times and higher power. The choice of method depends on factors such as the specific type of polysaccharide being extracted, the required purity, and the intended downstream applications (Bikmurzin et al., 2022).

### 4.2 Nonmechanical Extraction

The chemical, enzymatic, and physical extractions of polysaccharides from yeast cell wall are multistep processes, beginning with the disruption of yeast cells via enzymatic or chemical lysis to release intracellular contents. Enzymatic lysis employs specific enzymes, such as glucanases or zymolyase, to selectively degrade cell wall components, while chemical lysis uses reagents such as sodium hydroxide (NaOH) or detergents to dissolve cell walls and remove impurities (Boutros et al., 2022).

Autolysis is another option for extracting bioactive substances from yeast cells with high efficiency, although it may result in the loss of antioxidants including glutamine and vitamins such as folic acid (Jacob et al., 2019). Both autolysis and enzymatic decomposition share many similarities, with a few differences. The enzymes used in autolysis are endogenous, while those used in enzymatic decomposition are exogenous. Enzymatic decomposition relies on degrading cell wall proteins, which exposes the cell wall to osmotic shock (Takalloo et al., 2020). During autolysis, various intracellular materials, including proteins of various molecular weights (large, medium, and small), long‐chain and short‐chain fatty acids, and polysaccharides, are released into the culture medium simultaneously through passive transport processes (Wang et al., 2018).

The ionic liquid, known for its high polarity and low melting point, enhances the solubility of yeast β‐glucan, thereby increasing the extraction yield (Pham et al., 2020). For autolysis, NaCl is employed to create osmotic pressure, facilitating cell disruption (Xing et al., 2018). To extract high‐purity yeast β‐glucan from *S. cerevisiae*, the yeast is treated with the ionic liquid 1‐butyl‐3‐methyl‐imidazolium chloride resulting in yeast β‐glucan with a purity of 95.2% and a yield of approximately 83.4%. The mechanism involves the ionic liquid disrupting the cell walls and facilitating the release of yeast β‐glucan. Its high polarity enables interaction with the yeast β‐glucan, breaking hydrogen bonds and other interactions that typically stabilize the cell wall structure (Pham et al., 2020).

Once the cells are disrupted, the polysaccharides need to be solubilized. This can be accomplished through acid‐base extraction, where strong alkali solutions, such as HCl/NaOH, solubilize glucans and other polysaccharides (Divya et al., 2020). It has been reported that the alkaline pretreatment could attack the linkages within the polysaccharide chains, thus altering the degree of polymerization, surface area, and porosity, which can explain why sodium hydroxide pretreatment is considered an effective means for enzymatic hydrolysis (Zheng et al., 2019). Alkali treatment disrupts the spatial organization of yeast cell wall, releasing structural components but causing substantial losses of β‐1,3‐glucan. Consequently, alkali disaggregates and fragments yeast β‐glucan, reducing particle size, increasing surface area, and enhancing yeast β‐glucan solubility and enzyme accessibility (Zheng, Huang, Luo, et al., 2019). Yang and Huang (2021) reported that acid and alkali reagents are easy to corrode the equipment and pollute the environment. In addition, part of yeast β‐glucan will be degraded during the extraction process, and the stronger the acidity, the more obvious the degradation effect, thus reducing the yield and biological activity of the product.

Acid extraction, using acids like sulfuric acid (H_2_SO_4_), can hydrolyze certain polysaccharides selectively. Yeast β‐glucan extracted from *S. cerevisiae* using an acid‐base extraction method shows higher biological efficacy compared to water extraction, as reported by Amer et al. (2021). Kot et al. (2020) found acid hydrolysis to be the most effective cell wall disruption method compared to the ultrasound, osmotic shock, pasteurization, homogenization with zirconia balls, and freezing/defrosting. The washing step with acetic acid serves several crucial purposes: it removes impurities such as proteins, lipids, and other cellular components, ensures a purer glucan extract, maintains an acidic environment to preserve the glucan's structure, and prevents its degradation (Utama et al., 2020). In addition, the acid helps break down cell walls to release yeast β‐glucan from the cellular matrix and inactivates enzymes that could degrade glucan, thus preserving its integrity. Moderate acid extraction, such as with acetic acid, reduces the alkali‐insoluble β‐1,6‐glucan content but can also lead to β‐1,3‐glucan losses (Bzducha‐Wróbel et al., 2020).

The growth media has proven to be effective in enhancing the yield of yeast β‐glucan. Utama et al. (2020) successfully extracted yeast β‐glucan from *S. cerevisiae* using waste from bananas, papayas, and napa cabbage. Notably, the extraction process yielded the highest amount of yeast β‐glucan when papaya waste was utilized as the substrate. The *C. utilis*, cultivated on waste deproteinated potato juice water, was used for biomass production on a bioreactor scale (Bzducha‐Wróbel et al., 2019). The study highlighted that autoclaving yielded a higher β‐(1,3)/(1,6)‐glucan ratio and had a production efficiency of 70% compared to the alkaline‐acid procedure, indicating significant material losses in the alkaline‐acid process. Chotigavin et al. (2021) demonstrated that tannic acid plays a crucial role in enhancing yeast β‐glucan extraction from *Saccharomyces carlsbergensis*. By incorporating tannic acid into the yeast growing media, they achieved a significant increase in yeast β‐glucan content, reaching 42.23%. The mechanism proposed involves tannic acid forming complexes with yeast β‐glucan, which promotes their aggregation and precipitation, thus facilitating their extraction.

Yeast cell wall chitosan has some desirable properties, including lower molecular weights and higher degrees of deacetylation, especially suitable for biomedical applications (Islam et al., 2022). Araújo, Alves, Marques, et al. (2020) found that the chitin–glucan complex accounted for 20% dry mass (dm) of the cell from *K. pastoris* grown in glycerol. Aldebert et al. (2024) analyzed chitin patterns and morphology in 12 yeast species (including *Candida*, *Clavispora*, *Nakaseomyces*, *Pichia*, and *Saccharomyces*) using a lysis buffer and sonication steps. The extraction and characterization of chitin and chitosan from yeast cell walls, specifically from *S. cerevisiae* and *K. pastoris*, highlight distinct methods and yields. Using an alkaline‐acid extraction, *S. cerevisiae* produced chitin with a content of 92.80% (C. Sun et al., 2018). In contrast, Farinha et al. (2019) extracted a chitin–glucan complex from *K. pastoris*, constituting 19% of the biomass, followed by neutralization and dialysis. The binding of chitin with β‐1,3‐glucan and the high degree of polymerization of β‐1,3‐glucan contribute to its insolubility (Bikmurzin et al., 2022).

Autoclaving removes water‐soluble impurities, achieving production efficiency. This process effectively saponifies esterified lipids and removes proteins bound with mannan, though it results in considerable polysaccharide degradation (Bzducha‐Wróbel et al., 2020). Boiling alcohol is also used for lipid removal to reach higher purification of yeast β‐glucan (Bikmurzin et al., 2022; Bzducha‐Wróbel et al., 2020; Lowman et al., 2021). Hot water extraction involves boiling the cell wall material in water to solubilize polysaccharides. β‐Glucan were extracted from *Candida guilliermondii*, *Candida lusitaniae*, *Candida parapsilosis*, *P. pastoris*, *Saccharomyces pastorianus*, and *S. cerevisiae* using the hot water extraction method (Bikmurzin et al., 2022). This method produces yeast β‐glucan with fewer alkali‐insoluble polysaccharides but higher contamination with alkali‐soluble polysaccharides (Bzducha‐Wróbel et al., 2020).

Another physical method is cold plasma treatment, which degrades yeast cell walls through reactive oxygen species, reactive nitrogen species, ultraviolet photons, and charged particles. Siadati et al. (2020) demonstrated the impact of an Argon plasma jet on inactivating four different yeasts: *S. cerevisiae*, *Schizosaccharomyces pombe*, *C. parapsilosis*, and *Magnusiomyces magnusii*. The charged particles and electric fields generated during this process increase membrane permeability, leading to cellular leakage.

The enzymatic extraction of polysaccharides from yeast cell wall involves several steps starting with yeast cell preparation and disruption. Specific enzymes such as glucanases and proteases are used to hydrolyze the cell wall and release polysaccharides, targeting yeast β‐glucan and enhancing accessibility. Enzymes attack macromolecules without destroying yeast cell structure. Ultrasonication and alkaline pre‐treatments were used to reduce the particle size of yeast β‐glucan and enhance the efficiency of enzymatic hydrolysis (Zheng, Huang, Luo, et al., 2019). β‐Glucan with high molecular weight, high viscosity, and thermal stability can be obtained by the enzyme‐alkali extraction method because the protein content is greatly reduced by adding protease (Yang & Huang, 2021). Ma et al. (2022) utilized high‐pressure steam with neutral protease and papain for extracting yeast β‐glucan from *C. utilis*, improving solubility, reducing molecular weight, and enhancing free radical scavenging activity. Enzymatic hydrolysis is optimized by controlling pH, temperature, and incubation times to maximize efficiency.

The mechanical approaches result in the release of all the compounds of the yeast due to its non‐selectivity, which may prolong other steps, however, the low cost of the process and the ease of increasing the scale, the production of minimal waste, are among its benefits. While HPH and ultrasonication are effective, they can generate heat that might degrade heat‐sensitive polysaccharides. Cryogrinding and freeze‐thaw cycling are less likely to generate heat but may require longer processing times. On the other hand, non‐mechanical methods are more selective, but they can have destructive effects on components and nutrients.

### 4.3 Purification methods

The purification of polysaccharides extracted from yeast cell walls is a multifaceted process that combines various methods to achieve the desired purity. A combination of solvent precipitation, centrifugation, ultrafiltration, dialysis, and chromatography is commonly employed to remove impurities and refine the polysaccharides (Divya et al., 2020).

Physical methods such as centrifugation and ultrafiltration are integral to the purification process. High‐speed centrifugation separates components based on their size and density, effectively isolating polysaccharides from smaller, denser impurities. Pham et al. (2020) utilized filtration with hot water washing to separate yeast β‐glucan molecules based on their molecular weight, with purity of 95.2%.

Dialysis is commonly used to purify polysaccharides, where the extract is placed in a semi‐permeable membrane and immersed in water or a buffer solution, allowing small molecules and ions to diffuse out while retaining the larger polysaccharide molecules. The modified yeast β‐glucan from *S. cerevisiae* exhibited higher purity, likely due to the removal of proteinaceous and other non‐carbohydrate components during the dialysis step (Ishimoto et al., 2018). *P. pastoris* β‐glucan purity of 85.3% and a yield of 11.7% were obtained after high‐pressure pressure hot water, and ultrasound combined treatment followed by dialysis (Xing et al., 2018).

Chromatographic techniques, including size‐exclusion chromatography, ion‐exchange chromatography, and affinity chromatography, provide high specificity and purity (Zheng, 2019; Zheng, Huang, Ling, 2019). Size‐exclusion chromatography separates polysaccharides based on their size, ion‐exchange chromatography on their charge, and affinity chromatography on specific interactions with the stationary phase (M. Liu, Liu, et al., 2021). While these methods yield highly pure polysaccharide fractions, they are complex, costly, and require specialized equipment. In a study, the phosphorylated yeast β‐glucan was subjected to a rigorous purification process using an ion‐exchange chromatography column (Mei et al., 2020). This method efficiently isolated the desired compound, ensuring high purity and stability. In practice, a combination of these methods is often employed to achieve the highest purity. For example, Zheng, Huang, Ling (2019) purified the water‐soluble yeast β‐glucan sample using chromatography. After centrifugation and filtration, the filtrate indicated partial removal of zymoprotein. The final polysaccharide fraction, eluted with ultrapure water, had trace protein (1.0%).

Overall, the purification of polysaccharides from yeast cell walls requires a strategic combination of physical, chemical, and chromatographic techniques to achieve high purity, with each method contributing to the removal of specific impurities and the refinement of the final product for potential industrial and biomedical applications.

## 5 APPLICATIONS OF YCWPs

### 5.1 Encapsulation and structure modification material

This section highlights the diverse applications of yeast β‐glucan, including its roles in probiotic encapsulation, cryoprotection, bioactive compound delivery, hydrogel formation, and edible film development. These applications demonstrate the versatility of yeast β‐glucan in enhancing the stability and functionality of various products and processes. Table 3 shows the potential usage of the YCWPs as the features of creating structures for different purposes. β‐Glucan is highly effective for encapsulating probiotics due to its unique macroporous honeycomb structure (Gani et al., 2018). β‐Glucan is a promising prebiotic that selectively promotes the growth of beneficial gut bacteria, particularly *Lactobacilli* and *Bifidobacteria*, and its use in probiotic encapsulation is expected to enhance microorganism viability during gastrointestinal track, processing, and storage (Gani et al., 2018). da Silva Guedes et al. (2019) evaluated yeast β‐glucan as a cryoprotectant for probiotic *Lactobacillus* strains during freeze‐drying, storage, and in vitro digestion. Yeast β‐glucan and fructooligosaccharides significantly reduced viability loss compared to saline solution, with yeast β‐glucan maintaining higher probiotic counts and minimizing membrane damage. Yeast β‐glucan showed particularly strong protection for *L. plantarum* 201. The results suggest yeast β‐glucan is an effective cryoprotectant, warranting further research on its protective mechanisms and strain‐specific effects.

**TABLE 3:** Recent studies on the use of yeast cell wall polysaccharides (YCWPs) for various applications.

YCWPs	Application	Main findings	References
β‐Glucan isolated from brewer's spent yeast (*Saccharomyces uvarum*)	Cryoprotectant on the counts of *Lactobacillus acidophilus* 05, *L. plantarum 49*, and *L. plantarum 201*	Before and after freeze‐drying compared to saline solution (control): for *L*. *acidophilus* 05 and *L. plantarum 49* ↓ Viable cell counts for *L. plantarum 201* 120 days of storage at 4°C: ↑ Viable cell counts The membrane damage by β‐glucan and fructooligosaccharides compared to a saline solution (control), respectively: 21%–65.8% and 37.2%–83.5%	da Silva Guedes et al. (2019)
Commercial yeast β‐glucan	Encapsulation of apigenin with edible dock protein	Apigenin in β‐glucan ± Protein after intestinal digestion: ↓ Particle size from 2.3 µm to 261 nm compared to apigenin + protein (from 2.6 µm to 405 nm) ↑ Zeta‐potential: 33.16 mV compared to apigenin + protein (32.82 mV)	Zhou et al. (2024)
Commercial yeast carboxymethyl glucan	Encapsulation of resveratrol with or without zein	Encapsulation efficiency: Carboxymethyl glucan + zein: 85.4%, Carboxymethyl glucan: 38.0%, and zein: 72.7%. Resveratrol retention rate against light and heat: Carboxymethyl glucan + zein > carboxymethyl glucan > zein ↓ Loading capacity: Decreased for resveratrol‐zein+ carboxymethyl glucan (6.1%) compared to resveratrol + carboxymethyl glucan (9.5%) and resveratrol‐loaded zein (6.6%) Resveratrol release rate (simulated gastric digestion): Higher in resveratrol+carboxymethyl glucan compared to resveratrol‐loaded zein and resveratrol‐zein+ carboxymethyl glucan	Bao et al. (2023)
Chitin–glucan complex from *Komagataella pastoris* by the hot alkaline procedure (NaOH and KOH solutions, either at 1 or 5 mol/L)	Hydrogel	Hydrogel structure: More compact and denser via higher alkali concentration Stronger and more cohesive via higher polymer Higher elasticity via lower alkali concentration and KOH ↑ Ionic and mechanical strength, network density via NaOH Freeze‐thaw procedure aids the hydrogel formation	Araújo, Alves, Lima, et al. (2020)
Chitin–glucan complex from *Komagataella pastoris* by the hot alkaline procedure (NaOH and urea at different ratios)	Regenerated biopolymer via dialysis	Chitin Content: Increased with higher NaOH concentration. In regenerated polymers: ↓ Degree of acetylation, crystallinity index, thermal stability	Araújo, Alves, Marques, et al. (2020)
β‐Glucan from the brewer's spent yeast by alkaline‐acid method	Biofilm	↑ Solid content and overall thickness ↓ Dissolution time ↓ UV light (region: 200–400 nm) transmission The hydrophilic nature of β‐glucan facilitated the permeation of water vapor through the film The highest water vapor transmission rate and permeability via β‐glucan+ pomegranate juice	Avramia and Amariei (2023)

Abbreviations: ↑: upregulation; ↓: downregulation; β‐G: β‐Glucan.

Various studies have explored the encapsulation of yeast β‐glucan with a range of proteins and bioactive compounds, particularly those that are hydrophobic (Zhou et al., 2024). Apigenin, when loaded into yeast β‐glucan with edible dock protein nano micelles, improved its stability and bioavailability. This setup allowed apigenin to be released gradually, slowed its breakdown in stomach acid, and demonstrated great storage stability and compatibility with cells (Zhou et al., 2024). Bao et al. (2023) found that resveratrol in the composite particles of yeast carboxymethyl glucan and zein showed the highest encapsulation efficiency (85.4%) and stability for resveratrol, outperforming the individual usage of yeast carboxymethyl glucan and zein particles. They also delayed resveratrol release in simulated digestion, suggesting better oral bioavailability.

Hydrogels are 3D networks of molecules that can absorb and hold large amounts of water and other liquids, causing them to expand (Araújo, Alves, Marques, et al., 2020). Araújo, Alves, Lima, et al. (2020) examined the dissolution of the chitin–glucan complex from *K. pastoris* using NaOH and KOH solutions at 1 and 5 mol/L, followed by dialysis to form hydrogels. NaOH‐derived hydrogels were denser with smaller pores, exhibiting higher mechanical strength. In contrast, KOH‐derived hydrogels had larger pores and were more elastic. Rheological and texture analyses showed that NaOH‐based hydrogels, especially at higher concentrations, had superior mechanical properties. Another study by Araújo, Alves, Marques, et al. (2020) indicated that chitin–glucan complexes dissolve best in a solvent with high urea and low NaOH, with the regenerated polymers (dialysis of alkali solutions and lyophilization) showing reduced chitin content and thermal stability compared to the chitin–glucan complex (Araújo, Alves, Marques, et al., 2020).

Another potential application of yeast β‐glucan was explored by Avramia and Amariei (2023), who developed and examined yeast β‐glucan‐based films with pomegranate, bilberry, and cranberry juices. The inclusion of yeast β‐glucan in edible films increased their thickness and water vapor transmission rate due to their hydrophilic nature, especially in pomegranate juice films. It also enhances water vapor permeability and optimizes dissolution time for rapid solubilization in oral solutions. In addition, yeast β‐glucan contributed to the films' UV‐blocking properties, beneficial for protective packaging.

Yeast β‐glucan has been proven to be highly versatile, enhancing the effectiveness of probiotic encapsulation, cryoprotection, and bioactive compound stability, as well as improving mechanical and functional properties in packaging films. These advancements highlight its potential in various applications, warranting further research and development.

### 5.2 Functional food ingredient

YCWP as a functional food ingredient is summarized in Table 4. β‐Glucan is valued for its ability to gel‐forming and water‐holding capacity, which contribute to texture in food products. It can act as a thickener, fat replacer, stabilizer, and source of dietary fiber in a wide range of food matrices (Mejía et al., 2020). Fu et al. (2022) found that deproteinizing yeast β‐glucan reduced water‐holding capacity, but increased oil‐binding capacity and swelling power by removing hydrophilic proteins. Defatting boosted oil‐binding capacity and hypolipidemic properties but lowered swelling power. Freeze‐drying β‐Glucan preserved its water‐holding capacity, oil‐binding capacity, and enzyme‐inhibitory activities better than vacuum or hot air drying due to a more porous structure. In addition, Xu et al. (2022) showed that β‐glucan enhanced texture, gel strength, water‐holding capacity, and flavor‐binding sites in mackerel myosin gels, especially at high NaCl levels and with two‐step heating. These benefits were attributed to improved protein unfolding and denser gel networks, although β‐glucan slightly decreased gel whiteness without affecting acceptability. Overall, β‐glucan significantly improved myosin gel properties.

**TABLE 4:** Recent studies of yeast cell wall polysaccharides (YCWPs) as a functional food ingredient.

Yeast polysaccharide	Studied topic	Upregulated parameters	Downregulated parameters	References
β‐Glucan from the *S. cerevisiae* by autolysis‐hot water extraction	Functional properties	Deproteinization: oil‐binding capacity (39.4%) and swelling power (33.2%) Defatting: oil‐binding capacity (7.89%) Ball milling: oil‐binding capacity (20.8%) Freeze‐drying: water‐holding capacity, oil‐binding capacity, and swelling power	Deproteinization: water‐holding capacity (8.5%) Defatting: swelling power (8.85%) Ball milling: water‐holding capacity Hot‐air drying: water‐holding capacity, oil‐binding capacity, and swelling power	Fu et al. (2022)
Commercial yeast β‐glucan	Gelling properties with mackerel myosin	Hardness, springiness, gumminess, chewiness, resilience, Gel Strength, water holding capacity, elasticity (G'), and viscosity (G'')	Whiteness, bound, and free water mobility	Xu et al. (2022)
β‐Glucan from the *S. cerevisiae* by alkaline‐dialysis method	0.5% and 1% in yogurt Storage: 28 days, 4°C	Ash content, pH, firmness, compact microstructure, color change (yellowness, greenness, and darkness)	Syneresis, overall acceptability, sensory scores (appearance, flavor, texture, and overall acceptance)	Dos Santos et al. (2019)
β‐Glucan from the *S. cerevisiae* by alkaline‐acid method	0.1%, 0.5%, and 1% in skim milk yogurt Storage: 14 days, 4°C	Total acidity, probiotic activity, whey retention	pH, syneresis, flavor, and aroma (at higher concentrations)	Al‐Sahlany et al. (2022)
β‐Glucan from the yeast sediment of Viorica wine	0.1%, 0.2%, 0.3%, 0.4%, and 0.5% in skim milk yogurt	Titratable acidity, fermentation rate, viscosity, firmness of curd	pH, syneresis, coagulation time, whey separation	Chirsanova et al. (2021)
Commercial β‐glucan from the *S. cerevisiae*	0.025% in whey spread Storage: 28 days, 7°C	Ash, moisture, and protein (due to protein aggregation) content, yellowness, and greenness	Syneresis, consumer acceptance No obvious effect on sensory attributes and overall acceptability	Zanon et al. (2020)
β‐Glucan from the *S. cerevisiae* by acid, base, and other organic solvent extraction	1%, 2%, and 4% with or without 15% fat in refrigerated minced beef Storage: 11 days, 4°C	Shelf life, sensory attributes (color, flavor, tenderness, juiciness), overall acceptability	Free fatty acids, peroxide values, cholesterol levels	Msawil Al‐Shouki and Nasser (2019)
Commercial yeast β‐glucan	1%, 2%, 3%, 4%, and 5% in silvercarb surimi	Water‐holding capacity, reduction in fishy odor	Hardness, springiness, chewiness, and breaking force above 2% concentration	Zhang et al. (2019)
β‐Glucan from the brewer's spent yeast by alkaline‐acid method	0.8%, 1%, and 1.2% in bread	Water absorption rate, dough strength, viscosity, dough initial acidity	The moisture content of the dough and bread crumb, porosity, and specific volume of the bread	Marukhnenko et al. (2020)
Commercial β‐glucan from the brewer's spent yeast	0.2% and 0.4% in chilled bread Storage: 4 days, 4°C	Crumb hardness	Spread ratio, L* value	Suwannarong et al. (2019)
Commercial yeast β‐glucan	0.25%, 0.5%, 0.75%, 1%, and 2% in chilled bread Storage: 4 days, 4°C	Dough strength, adhesion, stickiness, moisture retention, specific volume, crumb texture	Dough elasticity, loaf volume, crumb structure, retrograded starch, staling	Suwannarong et al. (2020)
Commercial yeast β‐glucan	1.3%, 2.7%, and 4% substitution with 5%, 10%, and 15% of total fat in shortbread biscuit Storage: 22 days, 21°C	Moisture content, density, browning index	Spread ratio, L*, hardness, overall quality	Zbikowska et al. (2022)
Commercial yeast β‐glucan	1%, 2%, 3%, and 4% substitution with 20%, 40%, 60%, and 80% of total fat in muffin Storage: 14 days, 21°C	Hardness, crumb density, moisture retention	Volume, springiness, texture quality (sensory attributes)	Żbikowska et al. (2020)
β‐Glucan from the brewer's spent yeast by alkaline‐acid method	1%, 2.5%, and 4% in pasta	Dough hardness, resilience, water absorption, cooking loss	Dough stickiness and adhesiveness, cohesiveness, viscoelastic moduli (Gʹ and Gʺ), pasta firmness	Ungureanu‐Iuga and Avrămia (2024)

Abbreviations: ↑: upregulation; ↓: downregulation; β‐G: β‐Glucan.

β‐Glucan is widely used in the food industry, particularly in the dairy products, for its ability to enhance texture, bind moisture, and mimic the functionality of fat (Al‐Sahlany et al., 2022; Mykhalevych et al., 2022). Its application in yogurt has been shown to improve firmness and microstructure without affecting total solids, protein, or fat content (Dos Santos et al., 2019). Yeast β‐glucan also plays a significant role in lactose fermentation, reducing coagulation time, lowering pH, and increasing acidity, while simultaneously reducing whey syneresis and enhancing texture and stability (Al‐Sahlany et al., 2022; Chirsanova et al., 2021; Dos Santos et al., 2019). These properties make yeast β‐glucan an effective ingredient for improving rheology and viscosity in dairy products, often replacing gums including pectin, methylcellulose, and alginates at lower concentrations (Sengül & Ufuk, 2022). However, while yeast β‐glucan fortified yogurts offer improved texture, further research suggests that the palatability of these products needs to be enhanced to boost consumer acceptance (Dos Santos et al., 2019). Lower concentrations of yeast β‐glucan are preferred to maintain a desirable flavor and aroma (Al‐Sahlany et al., 2022; Dos Santos et al., 2019). Moreover, Zanon et al. (2020) demonstrated that adding yeast β‐glucan to whey protein spreads improved protein content and structural properties, although it did not significantly alter the protein, moisture, fat, or carbohydrate content, and had little effect on consumer acceptance compared to spreads without yeast β‐glucan. These findings suggest that yeast β‐glucan can be a valuable ingredient for improving both the functional and sensory properties of dairy products, though attention to flavor and aroma remains important for consumer preference.

Yeast β‐glucan also has the potential as a natural preservative and fat replacer in meat products, improving sensory attributes such as color, flavor, tenderness, and juiciness (Msawil Al‐Shouki & Nasser, 2019; Zhang et al., 2019). Msawil Al‐Shouki and Nasser (2019) found that adding yeast β‐glucan (1%, 2%, 4%) to minced meat tablets during refrigerated storage significantly reduced free fatty acids, peroxide values, and cholesterol levels, extending the shelf life and maintaining quality. Zhang et al. (2019) found that adding yeast β‐glucan to surimi gel at a 2% concentration significantly enhances its mechanical properties, water‐holding capacity, and sensory attributes by reinforcing the gel structure. However, concentrations above 2% disrupt the gel network, leading to a decline in these properties.

In bakery products, yeast β‐glucan improved the texture of bread, increasing its nutritional value of bread, as well as increasing the number of pores and fiber, causing better absorption of water by the dough (Martins et al., 2018; Suwannarong et al., 2019, 2020). Nevertheless, moderate yeast β‐glucan in bread and shortbread biscuits improved bread quality, while excessive amounts lead to undesirable texture and staling effects (Suwannarong et al., 2020; Zbikowska et al., 2022). Marukhnenko et al. (2020) discovered that adding yeast β‐glucan to dough increased water absorption by 4%, strengthened the dough, and increased water‐flour suspension viscosity. It slightly reduced dough moisture and increased acidity. In bread, yeast β‐glucan reduced crumb moisture and porosity without affecting taste or smell, and higher dosages led to lower moisture content and specific volume due to water absorption by yeast β‐glucan molecules. Suwannarong et al. (2019) studied the effects of yeast β‐glucan, extra water, and chilled storage on bread quality. They found that yeast β‐glucan decreased spread ratio and crumb lightness while increasing crumb yellowness and impacting moisture and texture. The optimal formulation was 0.28% yeast β‐glucan and 11.69% additional water, stored for four days, resulting in qualities comparable to fresh commercial bread and outperforming a basic formula. Another study by Suwannarong et al. (2020) found that adding up to 0.75% yeast β‐glucan to wheat flour dough improved its strength, adhesion, and elasticity, while higher levels (2%) had negative effects. Bread with 0.25%–0.75% yeast β‐glucan retained the moisture better and staled less, with 0.75% being optimal for volume and crumb texture. Higher levels lead to smaller loaf volume, denser crumb, and increased starch retrogradation. Ungureanu‐Iuga and Avrămia (2024) reported that yeast β‐glucan in pasta dough increased hardness and water absorption but reduced stickiness, cohesiveness, and firmness. It disrupted the gluten network, resulting in a less compact dough, higher cooking loss, and reduced viscoelastic properties.

Yeast β‐glucan has been investigated as a fat substitute in wheat‐based batter products such as shortbread biscuits and muffins, effectively reducing lipid content and energy value (Żbikowska et al., 2020; Zbikowska et al., 2022). Adding yeast β‐glucan resulted in denser shortbread biscuits with less spread during baking, as well as darkened the biscuits, decreased hardness. Storage increased hardness and moisture, particularly in microbial β‐glucan variants. Smaller fat substitutions had the least adverse impact on quality (Zbikowska et al., 2022). In muffins, yeast β‐glucan increased hardness and crumb density while reducing volume and springiness. Higher yeast β‐glucan content led to greater moisture retention but negatively impacted texture, flavor, and aroma, with quicker hardness increase and texture degradation during storage (Żbikowska et al., 2020).

Yeast β‐glucan improves texture, stability, and health benefits in various foods, enhancing dough strength and moisture retention while reducing lipid content. However, excessive yeast β‐glucan can negatively impact texture and quality, highlighting the need for optimal concentration and further research.

## 6 FUTURE DIRECTIONS, CHALLENGES, AND CONCLUSION

The production of YCWP from food waste offers substantial promise for sustainability and innovation in various industries, but it also faces significant challenges that need to be addressed before it can reach its full industrial and commercial potential.

- Optimization of food waste substrates: One of the primary challenges is the selection and optimization of food waste substrates for yeast cultivation. Food waste, while abundant and rich in nutrients, is highly variable in its composition depending on the source—agricultural byproducts, restaurant waste, or industrial food processing waste. This variability can affect yeast growth and polysaccharide yield. Certain food waste substrates, such as citrus peels, contain antimicrobial compounds such as terpenes and limonene, which can inhibit yeast fermentation. Pretreatment methods, such as autoclaving, chemical neutralization, or enzymatic detoxification, may be required to remove these inhibitors. However, these additional steps can increase both the complexity and cost of the overall production process, potentially offsetting the environmental and economic benefits of using food waste as a substrate.
- Economic viability and cost‐effectiveness: Another significant challenge is demonstrating the economic viability of YCWP production on a commercial scale. While food waste is a low‐cost raw material, the costs associated with fermentation, extraction, and purification processes can be substantial. Mechanical extraction methods, such as bead milling and HPH, are energy‐intensive and require expensive equipment. Nonmechanical methods, such as enzymatic or chemical extraction, although more selective and gentler, can be time‐consuming and costly due to the price of reagents and enzymes. To achieve widespread adoption, collaborative efforts between industry and academia are needed to optimize process efficiency, lower costs, and improve yield. Developing cost‐effective, scalable technologies that maintain product quality without excessive energy or resource input is essential for industrial success.
- Regulatory and safety approvals: Regulatory hurdles present a significant barrier to the commercial use of YCWP, particularly when derived from food waste. Before these products can be marketed for food, pharmaceutical, or cosmetic applications, they must undergo rigorous safety testing and receive regulatory approval from bodies such as the U.S. Food and Drug Administration (FDA) or the European Food Safety Authority (EFSA). Currently, some YCWP, like yeast β‐glucans, have been granted Generally Recognized as Safe (GRAS) status, but the use of chitin or chitosan derived from yeast cell walls remains under‐regulated. The FDA and EFSA have approved health claims for oat and barley β‐glucan related to cholesterol reduction and heart health, and 3 g or more per day of soluble β‐glucan fiber from whole oats, barley, or a combination of both is recommended (FDA, 2024; EFSA, 2011). As a result, many studies have focused on optimizing the extraction and characterization of β‐glucan from sources including oats, barley, and sorghum and have further driven its use in the food industry (Mejía et al., 2020). Cereal β‐glucans have similar structures but differ in their 1,3 to 1,4 linkage ratio and molecular size, and some contain large cellulose structures. Noncereal β‐glucans are found in yeast, fungi, bacteria, and algae, with yeast β‐glucans featuring linear (1,3) backbones and long (1,6) branches. Originally, noncereal β‐glucans have stronger immune‐boosting effects and are the main focus of immunomodulation and anticancer research. For this reason, yeast β‐glucan was used in dietary supplements and functional foods to support the immune system (Murphy et al., 2020). Achieving regulatory approval for food ingredients sourced from unconventional substrates, like food waste, requires detailed studies on the safety, toxicity, and allergenicity of these compounds. These studies can be costly and time‐intensive, creating another barrier to market entry.

The regulatory status of chitin and chitosan, however, remains more complex. While chitosan is widely recognized for its applications in dietary supplements, food preservation, and packaging materials, its direct use in food formulations is still under regulatory scrutiny. The FDA has evaluated chitosan for specific applications, including antimicrobial coatings; however, its broader use in food systems remains limited due to the need for further toxicological and allergenicity assessments. In contrast, the EFSA has approved chitosan hydrochloride derived from fungal sources as a novel food (EFSA Panel on Nutrition, 2011), demonstrating an increasing regulatory acceptance beyond traditional crustacean‐derived sources. In addition, EFSA has listed chitosan as an approved food additive to be used as a stabilizer and emulsifier in certain applications, although its use in food coatings and contact materials still requires case‐by‐case evaluation.

Yeast extract from brewers' spent yeast has been developed and used for 70 years, with large‐scale production worldwide. While new, high‐value uses are emerging, most yeast extract is still used in relatively lower‐value applications such as animal feed and microbial culture (Liu et al., 2021). Nonetheless, the FDA reported “baker yeast extract” as generally recognized safe (GRAS), and it can be used as a flavoring or helper agent in food, but not more than 5% of the total food amount (FDA, 2023). Although yeast β‐glucan can be derived from baker's yeast, brewers' spent yeast remains the most commercially significant source. The EFSA Panel on Dietetic Products & Nutrition & Allergies (NDA) (2011) concluded that yeast β‐glucan, derived from baker's yeast, is safe for use as a novel food ingredient in specified amounts for food supplements and other foods, based on its characteristics, production process, and available safety data. Furthermore, in 2011, the European Union approved the use of yeast β‐glucan as a food ingredient in a variety of foods including fruit drinks, cereal bars, biscuits, crackers, breakfast cereals, yogurt, chocolate, soups, and protein bars, but not in infant formula, and has also specified its maximum limits (European Union, 2011). Later, in 2017, the European Union expanded yeast β‐glucan usage for infant and young children's formulas (European Union, 2017). Moreover, producing yeast from food waste and subsequently extracting β‐glucan from these yeasts presents a sustainable alternative, potentially reducing waste while providing a valuable functional ingredient.

Beyond regulatory approvals, industry players are actively working to address regulatory challenges associated with food waste‐derived ingredients. Companies seeking to introduce chitin, chitosan, and other nontraditional food components are employing strategies such as presubmission consultations with regulatory bodies, third‐party safety evaluations, and participation in multi‐stakeholder initiatives to align with future regulatory trends. The Upcycled Food Association has also introduced a certification program, encouraging companies to proactively address regulatory concerns while promoting the sustainable use of food waste. Given the increasing interest in circular bioeconomy approaches, future regulatory frameworks may evolve to facilitate the integration of food waste‐derived materials into safe and approved food applications. Ongoing research, transparency in processing methodologies, and active regulatory engagement will be crucial to overcoming these challenges.

β‐Glucan is notable as a food ingredient for its roles as a texture regulator, fat substitute, and cryoprotective material for probiotics. Yeast β‐glucans, in particular, offer numerous health benefits, often surpassing those of cereal β‐glucans, as highlighted in the literature. Future regulatory frameworks should evolve to facilitate the integration of food waste‐derived materials into approved food applications. Ongoing research, transparency in processing methodologies, and proactive engagement with regulatory agencies will be crucial in overcoming these challenges.

(iv) Technological barriers in extraction and purification: The extraction and purification of YCWP present considerable technological challenges. Mechanical extraction methods, such as milling or homogenization, are effective at disrupting yeast cells but tend to be nonselective, releasing a mixture of cellular components that require extensive downstream purification. These additional steps can lengthen the process and reduce overall efficiency. Nonmechanical methods, including enzymatic extraction, offer more specificity but come with high costs and scalability issues. For instance, enzymes such as glucanases and zymolyases, used to degrade specific components of the yeast cell wall, can be expensive and require precise process control to avoid polysaccharide degradation.

Moreover, maintaining the bioactivity of polysaccharides during extraction is critical, especially when these compounds are intended for functional food or pharmaceutical applications. Heat‐sensitive compounds, such as β‐glucans, can degrade during high‐temperature mechanical processes. Achieving the balance between effective cell disruption and maintaining polysaccharide integrity is a key technological challenge. Developing innovative, low‐energy extraction methods, such as integrating bioreactors with advanced purification technologies, can enhance efficiency while maintaining the bioactivity of extracted polysaccharides.

(v) Impact on food matrices and product development: Incorporating YCWP into food products presents both technical and sensory challenges. β‐Glucans and chitin possess valuable functional properties, such as improving texture and acting as fat replacers, but at higher concentrations, they can negatively affect the sensory characteristics of food products. For example, high levels of β‐glucan in bakery products or dairy matrices may lead to undesirable changes in texture, flavor, or mouthfeel (Al‐Sahlany et al., 2022; Dos Santos et al., 2019). In addition, in fat‐reduction applications, β‐glucan may not fully replicate the mouthfeel and sensory experience provided by fats, requiring the development of sophisticated formulations to balance these effects.

Another challenge is understanding how YCWP behaves in different food matrices, especially during processing steps such as baking, fermentation, or pasteurization. These processes may alter the functional properties of β‐glucans or chitin, potentially diminishing their health benefits or altering their textural contributions. Future research should explore novel formulation techniques to optimize the balance between functionality and sensory attributes, ensuring consumer acceptability and maintaining product quality.

(vi) Consumer acceptance: Even if the technical and regulatory hurdles are overcome, consumer acceptance remains a critical challenge. Despite the growing interest in sustainable and functional ingredients, there may be resistance to food products derived from waste streams, as consumers could perceive them as inferior or unsafe. To gain consumer trust, companies must clearly communicate the environmental and health benefits of using food waste‐derived polysaccharides. In addition, marketing these products as clean‐label, natural, and health‐promoting ingredients will be essential in aligning with consumer trends toward sustainability and wellness.

While the production of YCWP from food waste offers considerable promise for contributing to a sustainable bioeconomy, it also faces substantial challenges that must be addressed. These include optimizing the use of food waste substrates, overcoming economic and technological barriers in extraction and purification, navigating complex regulatory frameworks, and ensuring consumer acceptance. Addressing these challenges will require interdisciplinary collaboration between researchers, industry, and policymakers to develop cost‐effective, scalable, and sustainable production processes. To gain consumer trust, industry stakeholders should implement targeted communication strategies that emphasize the environmental and health benefits of food waste‐derived polysaccharides. Educational campaigns and clean‐label marketing approaches will be essential in fostering positive consumer perception and market adoption.

While the production of YCWP from food waste offers considerable promise for contributing to a sustainable bioeconomy, it also faces substantial challenges that must be addressed. These include optimizing the use of food waste substrates, overcoming economic and technological barriers in extraction and purification, navigating complex regulatory frameworks, and ensuring consumer acceptance. To overcome these challenges, a collaborative effort between biotechnology companies, regulatory agencies, and academic researchers is essential. Industry stakeholders should work together to develop cost‐effective, scalable, and sustainable production processes while ensuring regulatory compliance and consumer acceptance.

A deeper understanding of the biosynthesis of YCWPs and the selection of appropriate food waste substrates are important factors in optimizing their performance and production. Future research should prioritize metabolic and genetic engineering approaches to enhance polysaccharide biosynthesis efficiency, optimize extraction processes, and identify novel applications for yeast‐derived biopolymers. In addition, interdisciplinary collaborations between biotechnology companies and academic researchers will be key in advancing production methods, conducting economic evaluations, and accelerating commercialization. By advancing technologies, securing regulatory approvals, and demonstrating economic viability, YCWP can potentially become a cornerstone in sustainable food and pharmaceutical industries, contributing to waste reduction, resource efficiency, and the development of innovative products.

## AUTHOR CONTRIBUTIONS

**Deniz Günal‐Köroğlu**: Writing—original draft; writing—review and editing. **Gulsah Karabulut**: Writing—original draft; writing—review and editing. **Fariddudin Mohammadian**: Conceptualization; writing—original draft. **Aslı Can Karaca**: Supervision; writing—review and editing. **Esra Capanoglu**: Supervision; writing—review and editing. **Tuba Esatbeyoglu**: Supervision; conceptualization; writing—review and editing.

## CONFLICT OF INTEREST STATEMENT

The authors declare no conflicts of interest.
